# Cost-Effectiveness of Therapist-Guided Internet-Based Cognitive Behavioral Therapy for Stress-Related Disorders: Secondary Analysis of a Randomized Controlled Trial

**DOI:** 10.2196/14675

**Published:** 2019-09-13

**Authors:** Elin Lindsäter, Erland Axelsson, Sigrid Salomonsson, Fredrik Santoft, Brjánn Ljótsson, Torbjörn Åkerstedt, Mats Lekander, Erik Hedman-Lagerlöf

**Affiliations:** 1 Division of Psychology Department of Clinical Neuroscience Karolinska Institutet Stockholm Sweden; 2 Center for Psychiatry Research Department of Clinical Neuroscience Karolinska Institutet Stockholm Sweden; 3 Stress Research Institute Stockholm University Stockholm Sweden; 4 Osher Center for Integrative Medicine Department of Clinical Neuroscience Karolinska Institutet Stockholm Sweden

**Keywords:** psychological stress, adjustment disorder, exhaustion disorder, cognitive behavioral therapy, cost effectiveness, internet

## Abstract

**Background:**

Stress-related disorders are associated with significant suffering, functional impairment, and high societal costs. Internet-based cognitive behavioral therapy (ICBT) is a promising treatment for stress-related disorders but has so far not been subjected to health economic evaluation.

**Objective:**

The objective of this study was to evaluate the cost-effectiveness and cost-utility of ICBT for patients with stress-related disorders in the form of adjustment disorder (AD) or exhaustion disorder (ED). We hypothesized that ICBT, compared with a waitlist control (WLC) group, would generate improvements at low net costs, thereby making it cost-effective.

**Methods:**

Health economic data were obtained in tandem with a randomized controlled trial of a 12-week ICBT in which patients (N=100) were randomized to an ICBT (n=50) or a WLC (n=50) group. Health outcomes and costs were surveyed pre- and posttreatment. We calculated incremental cost-effectiveness ratios (ICERs) based on remission rates and incremental cost-utility ratios (ICURs) based on health-related quality of life. Bootstrap sampling was used to assess the uncertainty of our results.

**Results:**

The ICER indicated that the most likely scenario was that ICBT led to higher remission rates compared with the WLC and was associated with slightly larger reductions in costs from pre- to posttreatment. ICBT had a 60% probability of being cost-effective at a willingness to pay (WTP) of US $0 and a 96% probability of being cost-effective at a WTP of US $1000. The ICUR indicated that ICBT also led to improvements in quality of life at no net societal cost. Sensitivity analyses supported the robustness of our results.

**Conclusions:**

The results suggest that ICBT is a cost-effective treatment for patients suffering from AD or ED. Compared with no treatment, ICBT for these patients yields large effects at no or minimal societal net costs.

**Trial Registration:**

ClinicalTrials.gov NCT02540317; https://clinicaltrials.gov/ct2/show/NCT02540317

## Introduction

### Background

Stress is considered to be one of the largest health challenges in the Western world [1]. Prolonged or repeated exposure to stress is associated with negative physical and mental health outcomes, decreased quality of life, and functional impairment [2,3]. The costs for employers and society are large because of high incidence of sickness absence, reduced productivity at work, and significant loss of potential labor supply [4]. For the afflicted individual, chronic stress can lead to high costs because of increased medical and insurance expenses and decreased income [1]. Taken together, the full scope of chronic stress can negatively impact a country’s gross domestic product, with conservative estimates of annual costs ranging from US $23 billion in the European Union [1] to US $42 billion in the United States [5].

When clinically significant symptoms and functional impairment result as a consequence of chronic or intermittent life stressors, diagnoses such as adjustment disorder (AD) or exhaustion disorder (ED) may be warranted [3,6]. AD is one of the most commonly used diagnoses by clinical psychologists and psychiatrists worldwide [7,8], cited by the Diagnostic and Statistical Manual of Mental Disorders, 5th edition (DSM-5) as being the principal diagnosis for 5% to 20% of mental health outpatients [9]. ED is a specification of the diagnostic category F43.8 (*other reactions to severe stress*) in the International Statistical Classification of Diseases and Related Health Problems, 10th edition [10] that has been accepted as a formal diagnosis by the Swedish National Board of Health and Welfare. Similar to the concepts of clinical burnout and allostatic overload [11,12], ED is characterized by severe mental and physical exhaustion, cognitive dysfunction, disturbed sleep, and somatoform complaints [3]. Compared with AD, ED is a more chronic and debilitating condition [13], but both are based on the longitudinal course of symptoms and behavioral changes in the context of stressful life events. Given the strain on health care systems to handle these stress-related disorders, health economic evaluations are important to provide decision makers with information about which treatments give maximum effect in relation to their cost [14]. Without knowledge of cost-effectiveness, there is a risk that health care resources are used inefficiently and that fewer can be offered treatment [15].

Cognitive behavioral therapy (CBT) is the most rigorously evaluated and effective psychological treatment for a range of mental health problems [16], including stress and stress-related symptoms [17,18]. To meet the high demand for CBT, delivering treatment via the internet has emerged as a viable option to increase treatment accessibility and reduce delivery costs. Therapist-guided internet-based CBT (ICBT) has been shown to be highly efficacious for many clinical conditions [19], often producing effect sizes in parity with face-to-face treatment while at the same time often requiring less than 10 min of therapist time weekly per patient [20]. A recent meta-analyses of Web- and computer-based interventions to reduce stress in diverse samples indicated that ICBT could be effective in reducing stress with small to moderate effect sizes [21]. Although these findings cannot be directly generalized to clinical populations suffering from stress-related disorders, we recently presented evidence that ICBT can also be effective in reducing symptoms in a clinical sample diagnosed with AD or ED [13].

Meta-analyses indicate that ICBT can be a cost-effective alternative for many clinical conditions [19,22]. Regarding interventions to reduce stress, however, health economic evaluations are scarce and generally suffer from low methodological quality [23]. A total of 2 recent studies have conducted health economic evaluations of internet-based stress-management interventions aimed at reducing stress in employees with elevated work-related stress [24,25]. Both the studies indicated that the interventions were cost-effective compared with waitlist control (WLC) conditions, from an employer’s [24] and a full societal perspective [25]. Although these results are promising, to the best of our knowledge, no study to date has investigated the cost-effectiveness of ICBT for patients actually diagnosed with stress-related disorders.

### Objectives

The aim of this study was to evaluate the cost-effectiveness and cost-utility of ICBT for stress-related disorders within the context of a randomized controlled trial [13], using both societal and health care perspectives. We hypothesized that ICBT, compared with a WLC group, would generate improvements at low net costs, thereby making the treatment cost-effective.

## Methods

### Design

**Figure 1:**
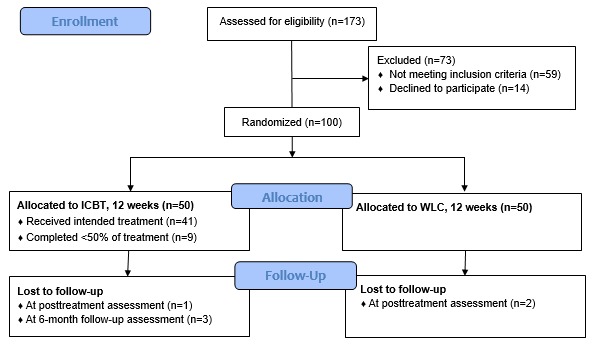
Flowchart illustrating trial design, adherence, and attrition. Note: The WLC was crossed over to treatment after the 12-week main phase of the trial and did not take part in the 6-month follow-up assessment. All health economic analyses are, therefore, based on the main phase of the trial. ICBT: internet-based cognitive behavioral therapy; WLC: waitlist control.

Health economic data were collected within a randomized controlled trial [13] in which adults suffering from stress-related disorders (N=100) were randomized to either an ICBT (n=50) or a WLC (n=50) group, each of 12 weeks duration. Stress-related disorders were defined as a clinical diagnosis of AD according to DSM-5 [9] or ED as defined by the Swedish National Board of Health and Welfare [3,10]. Randomization was stratified by diagnosis (AD vs ED) and took place after inclusion to prevent allocation bias. Health economic data were collected at pre- and posttreatment assessment (after 12 weeks) as well as at a 6-month follow-up (6MFU). As participants in the WLC were crossed over to treatment after 12 weeks, cost-effectiveness and cost-utility were assessed from the pretreatment to the posttreatment assessment only. However, data from the 6MFU are presented to allow for crude and uncontrolled estimates of stability in clinical outcomes and costs over time. The trial was conducted at Karolinska Institutet, Stockholm, Sweden, between September 2015 and August 2016. It was approved by the Regional Ethics Review Board in Stockholm, Sweden (2015/415-31/5) and preregistered at ClinicalTrials.gov (ID: NCT02540317). All participants provided verbal and written informed consent for study participation. Figure 1 illustrates the trial design and study flow.

### Study Sample

Detailed information about the recruitment procedure can be found in the study by Lindsäter et al [13]. The study was advertised through an ad in a national newspaper and through social media. Applicants were self-referred via a study Web portal, and primary inclusion criteria were (1) aged 18 to 65 years; (2) a primary diagnosis of AD or ED; (3) no substance abuse or dependence in the past 6 months; (4) no current or past psychosis or bipolar disorder; (5) no suicidal ideation; (6) if on medication with a monoamine agonist, this had been stable in the past month; and (7) no ongoing psychological treatment. Inclusion criteria were assessed by a licensed psychologist using information collected in a telephone-conducted clinical interview comprising the Mini-International Neuropsychiatric Interview [26] and data obtained from a Web-based screening. AD and ED were assessed with a clinical interview developed specifically for the trial that followed diagnostic criteria for respective diagnosis. The included sample consisted of 85% (85/100) women with a mean age of 47 (SD 8.8) years. A majority of patients (73%, 73/100) had a university education of 3 or more years, and 71% (71/100) of patients had a full-time employment at inclusion. Participants had, on average, suffered from their stress-related symptoms for 1.6 (SD 1.3) years. Patients with AD and ED were equally represented in the sample (53% [53/100] and 47% [47/100], respectively).

### Interventions

The CBT protocol used in the trial, on which this health economic study is based [13], has previously been tested in 2 randomized trials in the form of face-to-face therapy [27] and guided self-help [28] with promising results. A central component of the treatment was recovery training, which has previously been found to be important both in prevention and rehabilitation of chronic stress [29]. The treatment targeted increased recovery through scheduled recuperating activities and psychoeducation about sleep hygiene and stimulus control for improved sleep. Other central aspects of the treatment were behavioral activation [30] and exposure [31] to help patients break fear-avoidance patterns related to, for example, perfectionism, excessive worry, and assertiveness. Patients were encouraged to plan their days to create a balance between effortful expenditure and recovery.

The 12-week ICBT was delivered via a secure (encrypted traffic and 2-factor authentication) Web platform where psychoeducation, worksheets, and exercises were presented primarily through text. Patients received weekly written feedback on their work and progress by a licensed clinical psychologist who gave gradual access to the 12 treatment modules, similar to chapters of a book. Patients could contact their psychologist at any time and expect a reply within 48 business hours. Weekly automated text message reminders were sent to all patients in the ICBT group to log on to the treatment platform and work with the treatment. The average number of completed modules in ICBT was 9.2 (SD 3.2) out of 12, which is typically considered adequate and on par with many other ICBT protocols for common mental health conditions [32]. The mean therapist time per patient was 87 (SD 36) min, that is, on average, a little over 7 min weekly per patient.

Patients in the WLC group did not receive any treatment during the 12-week experimental phase and were encouraged not to make any changes in psychotropic medication or seek other psychological treatment for their stress-related disorder during that time. After 12 weeks, they were immediately crossed over to treatment.

### Clinical Assessment

For the cost-effectiveness analyses, effectiveness was defined as remission rate, estimated based on the 14-item Perceived Stress Scale (PSS) [33]. The PSS (which was administered online) measures how often one has perceived life as unpredictable, uncontrollable, and overloading in the past month. Patients respond on a 5-point response scale from 0 (never) to 4 (very often), with a total scale range of 0 to 56 (a higher score indicating higher level of perceived stress). The PSS has good construct validity and sensitivity to change in samples with stress-related disorders [34].

For the cost-utility analyses, the EuroQol 3L Questionnaire (EQ-5D) was used to assess health-related quality of life [35]. The EQ-5D is a nondisease-specific self-assessment questionnaire that measures 5 health domains: mobility, self-care, usual activities, pain and/or discomfort, and anxiety and/or depression [36]. An advantage of the EQ-5D is that an overall utility score of quality of life can be obtained, which can facilitate comparisons with trials using other health states and other disease conditions [37].

### Economic Evaluation

The economic evaluation framework of this study was a within-trial (12-weeks) cost-effectiveness and cost-utility analyses. Cost-effectiveness concerns the association between costs and efficacy (ie, remission rates on PSS), and cost-utility concerns the association between costs and health-related quality of life. Analyses were conducted from a societal perspective, including all direct and indirect medical costs (eg, health care consumption, costs for medicine, supplements, and alternative care/support) as well as nonmedical costs (eg, work cutback, domestic costs, and sick leave). We also conducted separate cost-effectiveness and cost-utility analyses from a health care perspective, based on direct medical costs only.

#### Measurement of Resource Utilization

To enable the estimation of costs, resource utilization data were obtained using the self-rated Trimbos and Institute Medical Technology Assessment of Costs Questionnaire for Psychiatry (TIC-P) [38]. This instrument measures resource use over the past month (except for medication use, which concerns the past 2 weeks). The TIC-P is used to estimate (1) direct medical costs (ie, health care utilization and medication costs), (2) direct nonmedical costs (eg, use of alternative medicine and medicaments), and (3) indirect nonmedical costs (costs associated with production loss generated by, for example, sick leave or work cutback due to presenteeism). The TIC-P has previously been shown to be a feasible and reliable instrument for collecting data on medical consumption and productivity losses [39]. All costs were initially assessed in the local currency (Swedish Krona or SEK) and converted into US Dollars ($) using 2016 as the reference year (yielding 1 SEK equivalent to US $0.1148). Scoring of the TIC-P and transformation of TIC-P scores to costs was done by the first author who, during this process, was blind to patient condition (ICBT vs WLC).

#### Estimation of Costs

We used public health care tariffs to estimate medical cost (see Multimedia Appendix 1). For each patient, tariffs were multiplied by the corresponding number of health service units (eg, consultations and sessions) from the TIC-P. Costs for medications (prescription and otherwise) were estimated using market prices in Sweden. As the TIC-P only asks for medication use in the past 2 weeks, these costs were multiplied by 2 to represent a time frame of 1 month. The human capital approach was used to estimate indirect costs of sick leave, unemployment, and work cutback, meaning that lost gross earnings were taken as a measure of production loss [37]. Salaries were estimated based on the average monthly earnings in Sweden, stratified by the level of education and gender, as listed by Statistics Sweden for the year 2015. To estimate costs for work cutback, participants reported the number of days worked when ill in the past month and an inefficiency score of how much productivity was reduced as compared with productivity when in good health. Reduced monthly productivity (based on days worked when ill) was multiplied with the percentage of production loss (inefficiency score) and with the estimated daily earning [40]. Costs of domestic work cutback and informal care from family and friends was estimated to US $10 per hour based on data from the study by Smit et al [41], an estimate that has been used in several recently published health economic trials [42,43]. Owing to the short period under study (12 weeks), we did not discount costs.

Direct medical costs associated with the intervention mainly comprised the clinician’s time for the 12 weeks of ICBT. Clinician time spent was logged for every contact with a patient and included reviewing the patient’s work and giving written feedback. As clinicians in this study were exclusively licensed psychologists working at a primary health care clinic, we used the cost tariff for a 45-min session with a psychologist within primary health care (US $103) multiplied by the clinician time for each individual patient.

### Statistical Analysis

All analyses adhered to the intention-to-treat principle, meaning that all randomized participants were eligible for analysis. Owing to the low degree of data loss (1 out of 50 in ICBT and 2 out of 50 in the WLC post treatment), no imputation of missing data was deemed necessary (we employed listwise deletion). Analyses were conducted in SPSS 20.0 (IBM) and Stata/IC 14.2 (Stata Corporation).

#### Analysis of Remission Rates

Remission was operationalized as clinically significant improvement on the primary outcome PSS. This meant that patients were required to (1) make a reliable change in accordance with the Jacobson and Truax [44] criteria (ie, a reduction of 7 units on the PSS) and (2) have a posttreatment rating on PSS that was closer to a normal population than to a clinical population. On the basis of the data from the study by Lavoie and Douglas [45], the postrating cutoff was defined as a PSS score <31. We analyzed differences in remission rates pre- to posttreatment using chi-square tests.

#### Analysis of Health-Related Quality of Life

The answers given in EQ-5D were combined to generate a utility score of health states ranging from 0 to 1, with 0 representing death and 1 representing full health [36], based on the Swedish experience-based time trade-off value set [46]. Owing to the relatively short time perspective (12 weeks), we used the difference in utility scores between the pre- and posttreatment assessment to measure the effect in terms of health-related quality of life in the different cost-utility analyses. To generate a frame of reference, we also conducted a separate cost-utility analysis from a societal perspective in which the utility scores were converted to quality-adjusted life years (QALYs) gained over the 12-week treatment phase. As utility scores demonstrated skewness and kurtosis of residuals, differences between the ICBT and the WLC were analyzed using bootstrapped linear mixed models (5000 replications) where time (pre-to posttreatment), group (ICBT vs WLC), and the time × group interaction were independent variables.

#### Analysis of Cost Changes

As in the case of utility scores, cost residuals were skewed and showed evidence of high kurtosis. We, therefore, analyzed between-group changes in costs (gross and net total costs as well as changes in specific cost-domains) from pre- to posttreatment using bootstrapped linear mixed-models (5000 replications).

#### Analysis of Cost-Effectiveness and Cost-Utility

To estimate cost-effectiveness and cost-utility, we used the incremental cost-effectiveness ratio (ICER) and the incremental cost-utility ratio (ICUR), respectively. These were calculated by taking the net cost difference between conditions (ICBT vs WLC) at posttreatment compared with baseline, divided by the difference in remission rate (ICER) or utility improvement (ICUR) between the groups over the 12-week treatment period [14]. In the analysis based on QALYs, the denominator of the ICUR was the ICBT versus WLC difference in per capita QALYs over the 12-week treatment period.

#### Assessment of Uncertainty

As ICER and ICUR point estimates are difficult to interpret [14], we modeled the uncertainty of these based on bootstrapping (5000 samples) of the treatment group’s incremental costs and effects (remission rate and utility) as compared with the WLC. The bootstrapped values then formed the basis for cost-effectiveness and cost-utility planes, with effects on the x-axis and costs on the y-axis. Cost-effectiveness and cost-utility planes allow a probabilistic decision-making approach [47], where a majority of ICERs or ICURs in the southeast quadrant of these planes indicate a larger effect of ICBT at a lower cost compared with the WLC, and a majority of ICERs or ICURs in the northeast quadrant suggest a larger effect of ICBT at a higher cost compared with the WLC. In the latter case, the amount of money a decision maker is willing to pay for, for example, 1 additional patient in remission, is crucial in determining whether a new treatment is to be adopted or not. Hence, we investigated the probability of the treatment being cost-effective compared with the WLC at a range of different willingness to pay (WTP) scenarios. WTP for an additional responder can be illustrated by the means of cost-effectiveness and cost-utility acceptability curves [48].

Additional sensitivity analyses were conducted to further confirm the robustness of results. First, we replaced the intervention cost for each patient in the ICBT group with US $663 (ie, more than thrice the mean intervention cost), based on an estimate of the cost of running ICBT in a psychiatric clinic [49]. The new intervention cost included an average therapist time for psychiatric patients in ICBT and costs for assessment procedures, referral and follow-up visits, other health care staff, hospital space, and platform administration [49]. Second, as previous investigations have shown that patients with AD and ED differ in symptom severity and functional disability [13], we calculated ICERs separately for the diagnostic groups and also constructed cost-effectiveness planes and acceptability curves from a societal perspective. Third, as costs for domestic work and informal care from family and friends are difficult to estimate [49], we calculated ICERs and modeled cost-effectiveness planes using 2 different scenarios: (1) a scenario in which we used an updated cost-estimate of US $19 (ie, almost doubling the cost-estimate used in the main analyses), representing the average gross hourly wage earned by a domestic worker as suggested by Bock et al [49], and (2) another scenario in which we removed the costs for domestic work and informal care altogether. Finally, although there are indications that production loss because of presenteeism is a major cost driver for a range of disease conditions, comprising on average 52% of total costs [50], there is to date no consensus regarding methods to accurately measure and value it. We, therefore, conducted a sensitivity analysis with the purpose of illuminating to what extent cost related to work cutback may impact the total cost-effectiveness of ICBT in our trial. We calculated ICERs and modeled cost-effectiveness planes from a societal perspective, both when excluding work cutback as a cost-domain and when doubling these costs.

## Results

### Data Completion and Receiving Treatment Outside of the Study

There was a 100% (100/100) data completion at pretreatment. At posttreatment assessment, data completion was 98% (49/50) in the ICBT group and 96% (48/50) in the WLC. At the 6MFU, 94% (47/50) of patients in the ICBT group completed assessments. A total of 3 patients (6%) in ICBT and 4 (8%) in the WLC reported having received other treatment for stress-related problems during the intervention period. Fisher exact test revealed no significant difference between the groups in this regard (*P=*.47). In the ICBT group, 2 patients received psychological treatment and 1 initiated psychotropic medication. In the WLC, 2 patients received psychological treatment and 2 initiated psychotropic medication.

### Clinical Efficacy: Remission Rates and Utility

Table 1 shows means and SDs at pretreatment, posttreatment, and 6MFU for PSS and utility scores. In this study, remission rate on PSS was the main clinical outcome. As previously reported in the main outcome study [13], 31 of 50 patients (62%) in the ICBT group were in remission (ie, met criteria for clinically significant improvement) post treatment, as compared with 5 of 50 patients (10%) in the WLC group, which was a statistically significant difference (X^2^_1_=29.3; *P*<.001). At the 6MFU, 34 of 50 patients (68%) in the ICBT group were in remission. Regarding utility, patients in the ICBT group significantly increased their health-related quality of life from pre- to posttreatment (beta=.05; 95% CI 0.03 to 0.08; *Z*=3.91; *P*<.001), whereas patients in the WLC group did not (beta=.02; 95% CI −0.01 to 0.05; *Z*=1.41; *P*=.16). However, there was no significant group × time interaction effect on utility from pre- to posttreatment (beta=.03; 95% CI −0.01 to 0.07; *Z*=1.68; *P*=.09). There was no significant change from posttreatment to 6MFU in utility in the ICBT group, indicating stability of improvements (beta=.01; 95% CI −0.02 to 0.04; *Z*=0.51; *P*=.61).

**Table 1:** Statistical values for primary outcome measure and health-related quality of life.

Measure and group		Pretreatment, mean (SD)	Posttreatment, mean (SD)	6-month follow-up, mean (SD)
**Perceived Stress Scale**				
	ICBT^a^	37.2 (7.1)	24.2 (8.6)	21.9 (7.7)
	WLC^b^	36.4 (7.3)	33.2 (7.9)	—^c^
**Utility^d^**				
	ICBT	0.82 (0.12)	0.87 (0.09)	0.89 (0.09)
	WLC	0.82 (0.11)	0.84 (0.11)	—

^a^ICBT: internet-based cognitive behavioral therapy.

^b^WLC: waitlist control.

^c^Not applicable. Patients in the WLC group were crossed over to treatment after the posttreatment assessment.

^d^Utility scores are based on EuroQol 3L Questionnaire health states.

### Cost Changes

Table 2 presents the per capita costs at the pre- and posttreatment assessment (for spatial reasons, costs at the 6MFU are presented in the Multimedia Appendix 1). There was no significant difference between groups from pre- to posttreatment regarding gross total costs (ie, societal costs excluding the intervention costs; beta=−260.22; 95% CI −877.91 to 357.47; *Z*=−0.83; *P*=.41) or net total costs (ie, societal costs including the intervention costs; beta=−57.61; 95% CI −685.67 to 570.45; *Z*=−0.18; *P*=.86). Analyses of specific cost-domains revealed a significant interaction effect between time (from pre- to posttreatment) and group (ICBT vs WLC) regarding costs related to work cutback, where patients in the ICBT group significantly reduced their costs compared with those in the WLC group (beta=−292.24; 95% CI −525.98 to −58.50; *Z*=−2.45; *P*=.014). No other between-group interaction effects were found in specific cost-domains based on the TIC-P. Gross total costs in the ICBT group remained stable between posttreatment and the 6MFU (beta=173.76; 95% CI −383.98 to −731.46; *Z*=0.62; *P*=.54).

**Table 2:** Per capita costs at pre- and posttreatment assessment (all costs are in US dollar, converted from the Swedish Krona).

Cost-domains		Pretreatment^a^				Posttreatment			
		ICBT^b^, (n=50)		WLC^c^, (n=50)		ICBT (n=49)		WLC (n=48)	
		Mean (SD)	Median	Mean (SD)	Median	Mean (SD)	Median	Mean (SD)	Median
**Direct medical**		187 (320)	6	316 (547)	10	172 (255)	41	350 (845)	213
	Health care visits	169 (288)	0	312 (546)	0	166 (254)	0	345 (845)	211
	Medication	18 (83)	2	4 (5)	1	6 (9)	2	5 (7)	3
Direct nonmedical		80 (120)	20	160 (445)	35	78 (130)	0	109 (173)	5
**Indirect nonmedical**		1115 (1436)	579	1043 (1629)	132	884 (1348)	313	1040 (1368)	515
	Unemployment	150 (746)	0	80 (568)	0	182 (910)	0	0 (0)	0
	Sick leave	389 (980)	0	639 (1406)	0	438 (929)	0	624 (1276)	0
	Work cutback^d^	408 (636)	149	221 (507)	0	212 (421)	0	317 (463)	178
	Domestic	168 (397)	56	103 (189)	26	53 (78)	13	99 (155)	31
Gross total costs		1382 (1533)	843	1518 (2080)	466	1134 (1528)	492	1499 (1841)	762
Intervention costs		—^e^	—	—	—	203 (78)	205	—	—
Net total costs		1382 (1533)	843	1518 (2080)	466	1314 (1553)	721	1499 (1841)	762

^a^There were no significant differences between groups (ICBT vs WLC) in any cost-domain at the pretreatment assessment (*P*=.06-.57).

^b^ICBT: internet-based cognitive behavioral therapy.

^c^WLC: waitlist control.

^d^Significant interaction effect between groups (ICBT vs WLC) from pre- to posttreatment (*P*=.014).

^e^Not applicable.

### Societal Perspective: Cost-Effectiveness and Cost-Utility

**Figure 2:**
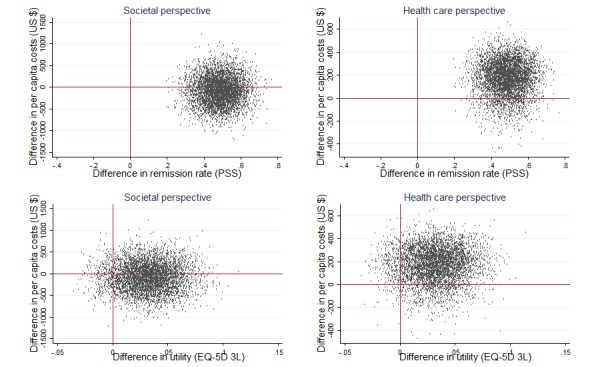
Cost-effectiveness (top) and cost-utility (bottom) planes comprising 5000 bootstrapped incremental cost-effectiveness ratios and incremental cost-utility ratios, respectively, comparing internet-based cognitive behavioral therapy with waitlist control over the 12-week treatment period. Remission was operationalized as the proportion of patients who made a clinically significant improvement on the 14-item Perceived Stress Scale (PSS). Utility was based on EuroQol Questionnaire, 3L version (EQ-5D 3L) health states. In the 2 left-hand planes, all costs from a societal perspective are included. The 2 right-hand planes include costs from a health care perspective (ie, using only direct medical costs). Please note that the y-scale of the health care perspective graphs differs from that of the societal perspective graphs.

Taking all costs into account (ie, conducting the analysis from a societal perspective), the ICER was −77.24/0.49=−157.63, favoring ICBT over the WLC. This indicates that the most likely scenario was that ICBT led to higher remission rates compared with the WLC and was associated with slightly larger reductions in costs from pre- to posttreatment. Figure 2 (top left) presents the scatter plot of simulated ICERs across the 4 quadrants of the cost-effectiveness plane, illustrating the degree of uncertainty associated with the estimated parameter. A majority of the simulated ICERs (60.86%, 3043/5000) are located within the southeast quadrant (larger effect of ICBT at a lower cost), whereas 39.14% (1957/5000) are located in the northeast quadrant (larger effect of ICBT at a higher cost). This suggests that ICBT is a cost-effective treatment from a societal perspective.

To estimate the cost-utility of ICBT relative to the WLC, we repeated the analysis using health-related quality of life (change in utility) as outcome. The ICUR was −77.24/0.03=−2574.67. As illustrated in Figure 2 (bottom left), a majority (57.20%, 2860/5000) of the simulated ICURs are located within the southeast quadrant, indicating that the most likely outcome is that ICBT, in comparison with no treatment, leads to lower net costs while increasing health-related quality of life. When QALYs were instead used as outcome, the ICUR was −77.24/0.0043=−17962.79. Of the simulated ICURs, 48.14% (2407/5000) fell in the southeast quadrant of the cost-utility plane, 34,52% (1726/5000) fell in the northeast quadrant, 11.90% (595/5000) fell in the northwest quadrant, and 5.44% (272/5000) in the southwest quadrant (see Multimedia Appendix 1 for cost-utility plane). This suggests that the most likely scenario is that the treatment generates more QALYs at lower net costs compared with no treatment.

**Figure 3:**
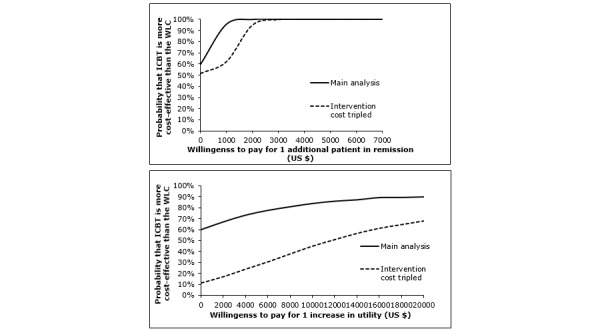
Cost-effectiveness (above) and cost-utility (below) acceptability curves from a societal perspective comparing internet-based cognitive behavioral therapy (ICBT) with waitlist control (WLC) over the 12-week treatment period. Note: Solid lines represent the probability of ICBT being more cost-effective in the standard scenario. Dotted lines represent the probability of ICBT being more cost-effective based on a sensitivity analysis in which a 3-fold higher intervention cost was used (US $663).

Figure 3 illustrates the acceptability curves based on the same data as used above in the cost-effectiveness and cost-utility analyses (using change in utility) from a societal perspective. As can be seen, ICBT has a 60% probability of being cost-effective from pre- to posttreatment if society is willing to pay US $0 for 1 additional case of remission or for increased utility. This was true also when using QALYs as outcome (see Multimedia Appendix 1). If society instead were willing to pay US $1000 for 1 additional case of remission, the probability of the treatment being cost-effective would increase to 96% (Figure 3, top). Assuming a WTP of US $8000, the probability of ICBT being cost-effective with regard to increasing quality of life would increase to 80% (Figure 3, bottom). Using QALYs as outcome, a WTP of US $25,000 and US $50,000 would increase the probability of 1 QALY gain in ICBT, compared with the WLC, to 71% and 79%, respectively.

### Health Care Perspective: Cost-Effectiveness and Cost-Utility

**Figure 4:**
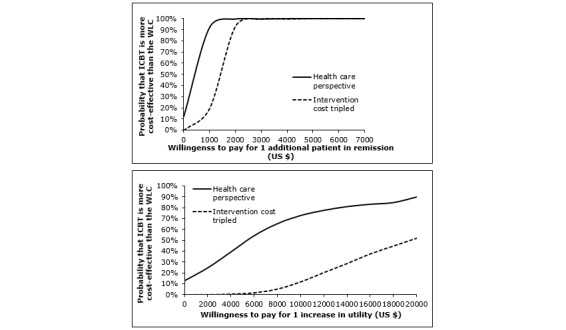
Cost-effectiveness (above) and cost-utility (below) acceptability curves from a health care perspective comparing internet-based cognitive behavioral therapy (ICBT) with waitlist control (WLC) over the 12-week treatment period. Note: Solid lines represent the probability of ICBT being more cost-effective in the standard scenario. Dotted lines represent the probability of ICBT being more cost-effective based on a sensitivity analysis in which a 3-fold higher intervention cost was used (US $663).

Taking only the direct medical costs into account, that is, a health care perspective, analysis using remission rate as efficacy measure generated an estimated ICER of 171.12/0.49=349.00. This means that each additional case of remission in ICBT relative to the WLC was associated with a slight increase in health care costs in the ICBT compared with the WLC. When conducting the same analysis using change in utility as outcome, the corresponding ICUR was 171.12/0.03=5704. Figure 2 shows the scatter plots of the simulated ICERs (top right) and ICURs (bottom right) from a health care perspective. Although treatment benefits of ICBT are associated with a cost for the health care provider, the cost-effectiveness acceptability curve (Figure 4, top) shows that, at a relatively low WTP of US $1000, the probability of ICBT being cost-effective from a health care perspective is 97%. Regarding cost-utility from a health care perspective, the ICBT has a higher probability of cost-utility compared with the WLC at a WTP of approximately US $6000 (Figure 4, bottom).

### Sensitivity Analyses

In the sensitivity analysis in which an intervention cost of US $663 (including, eg, assessment procedure, platform administration, and other health care staff and overhead costs) replaced the intervention cost of ICBT in this study, ICBT remained more likely to be cost-effective from a societal perspective compared with the WLC even at a WTP of US $0 (see Figure 3, top, dotted line). The cost-utility acceptability curve (Figure 3, bottom, dotted line) indicates that a WTP of approximately US $17,000 would be required for the ICBT to be preferable to the WLC in terms of cost-utility. From a health care perspective (see Figure 4, dotted lines), the new intervention cost estimate would require a WTP of approximately US $1500 for ICBT to be more likely than the WLC to be cost-effective from pre- to posttreatment. To render cost-utility of ICBT, compared with the WLC, from a health care perspective, a WTP of US $20,000 would be needed.

**Figure 5:**
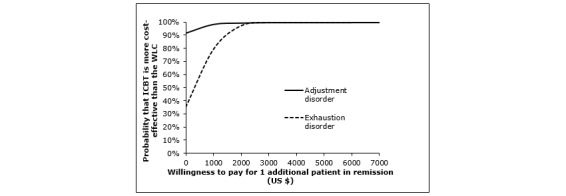
Cost-effectiveness acceptability curve from a societal perspective, comparing patients with adjustment disorder and exhaustion disorder in internet-based cognitive behavioral therapy (ICBT) to their respective wait-list control (WLC) over the 12-week treatment period. Remission is operationalized as clinically significant improvement on the Perceived Stress Scale (PSS).

Figure 5 illustrates the cost-effectiveness acceptability curves for the respective diagnostic groups (AD and ED), using remission rate on PSS as efficacy measure. For patients diagnosed with AD, there was a 92% probability that ICBT was more cost-effective than the WLC at a WTP of US $0. For patients diagnosed with ED, there was an 80% probability that ICBT was more cost-effective compared with the WLC at a WTP of US $1000. More information can be found in Multimedia Appendix 1 about outcomes, costs, and cost-effectiveness planes for AD and ED respectively (Multimedia Appendix 1).

The sensitivity analyses investigating different cost scenarios for informal care and domestic work indicated that the overall cost-effectiveness of ICBT remained unchanged when a higher cost-estimate was used and when costs for informal care and domestic work were removed from the total net costs. In the first scenario (where US $19 was used as a cost estimate of the average gross hourly wage earned by a domestic worker), the ICER was −126.69/0.49=−258.55 in favor of the ICBT, with 64.96% (3248/5000) of simulated ICERs falling in the southeast quadrant of the cost-effectiveness plane and 35.04% (1752/5000) falling in the northeast quadrant. In the second scenario (where costs for informal care and domestic work were removed), the ICER was −37.91/0.49=−1.99, supporting the superiority of ICBT with 55.10% (2755/5000) of simulated ICERs in the southeast quadrant and 44.90% (2245/5000) in the northeast quadrant (see Multimedia Appendix 1 for cost-effectiveness planes). Regarding presenteeism costs, the ICER in which costs for work cutback were removed indicated that each patient in remission was associated with a higher cost in the ICBT compared with the WLC (ICER: 214.43/0.49=437.61), with only 27.02% (1351/5000) of simulated ICERs in the southeast quadrant and 72.98% (3649/5000) in the northeast quadrant. At a WTP of US $2000, however, the probability of ICBT being cost-effective increased to 98% (see Multimedia Appendix 1). When costs for presenteeism were doubled, the resulting ICER was −368.91/0.49=−752.8 in favor of the ICBT, with 86.92% (4346/5000) of simulated ICERs in the southeast quadrant and 13.01% (654/5000) in the northeast quadrant, indicating that this scenario would render the treatment highly cost-effective as compared with the WLC. This sensitivity analysis supports that the net difference in these costs is largely in favor of the ICBT and that productivity loss because of presenteeism to a large extent contributes to the high cost-effectiveness of the treatment.

## Discussion

### Principal Findings

To our knowledge, this is the first health economic evaluation conducted of an ICBT specifically targeting stress-related disorders in the form of AD and ED. The results showed that ICBT generated higher remission rates compared with the WLC at no additional societal costs. The cost-utility analysis also showed that the ICBT was more likely to be preferable to the WLC from a societal perspective, both when change in utilities and QALYs were used as outcome. Taking a health care perspective (ie, including only direct medical costs in the ICER), there was a 97% probability that ICBT would generate an additional patient in remission compared with the WLC at a WTP of US $1000. A WTP of approximately US $6000 would, however, be required for the ICBT to be preferable to the WLC in terms of cost-utility. The overall indication of treatment cost-effectiveness was supported by sensitivity analyses. In sum, using data from a randomized controlled trial, this study showed that ICBT for stress-related disorders is likely to be a cost-effective treatment compared with no treatment.

The results of our study are in line with other health economic evaluations of ICBT for a range of clinical conditions, where ICBT in general has more than 50% probability of being cost-effective compared with no treatment or conventional CBT at a WTP of US $0 for an additional improvement [19]. As no previous health economic evaluations of ICBT for patients diagnosed with stress-related disorders have been conducted, to our knowledge, there are no available cost-effectiveness estimates of the target population with which we can directly compare our results. Nevertheless, 2 recently published studies evaluated cost-effectiveness of ICBT for employees with elevated levels of stress, 1 from an employer’s perspective (including only costs relevant for the employer, ie, presenteeism, absenteeism, and treatment costs) [24] and 1 adopting a full societal perspective [25]. Results of these studies were similar to those found in this study, with probabilities of treatment cost-effectiveness of approximately 70% compared with WLC at a WTP of US $0 [24,25]. In these studies, as in ours, costs because of presenteeism were large and constituted central cost drivers in the cost-effectiveness analyses. These clinical findings support previous reports stating that presenteeism constitutes a large cost in stress-related mental illness and many other clinical conditions [1,50], in fact reducing output by at least as much as absenteeism [50,51]. The indication that ICBT may significantly reduce costs within this domain is encouraging and needs to be further investigated. Although the potential cost savings related to reduced work cutback after ICBT may not directly benefit the health care provider (the payer of the intervention in this study), the low intervention cost and high scalability of ICBT makes the treatment cost-effective also from a health care perspective.

Although this study showed that ICBT for stress-related disorders is likely to be cost-effective, the degree of cost-effectiveness and cost-utility versus the WLC was, to some extent, moderated by 3 factors. First, the ICER was slightly more favorable when using a societal perspective compared with a narrower health care perspective. This is reflective of the absence of effect of ICBT on direct medical costs (health care visits and medication), which may be explained by the fact that patients suffering from chronic stress often have comorbid health conditions [52] that ICBT would be unlikely to affect in the short term. Second, the ICER (based on remission rates) was somewhat more favorable than the ICUR (based on utility change or QALYs). This was because the controlled effect on the primary outcome PSS was substantially larger than that on EQ-5D. This is expected given the generic nature of EQ-5D, which includes items such as “I am unable to wash and dress myself. *”* Third, even if ICBT was cost-effective for both diagnostic groups, it was more so for patients with AD compared with those with ED. These results can be understood by the fact that, relative to their respective controls, both diagnostic groups made large symptomatic improvements, but cost changes were smaller for patients with ED compared with patients with AD. A possible conclusion that can be drawn is that it is likely beneficial to offer treatment early on in the development of chronic stress (ie, for patients suffering from AD as opposed to ED), as a means of optimizing treatment cost-effectiveness and preventing increased societal costs. Nonetheless, it is encouraging that ICBT is cost-effective even in the very short term also for patients suffering from severe symptoms of chronic stress.

### Limitations and Strengths

There are some limitations to this study. First, the use of a WLC that (for ethical reasons) received treatment immediately after the 12-week experimental phase did not allow for between-group comparisons of costs and effects at the 6MFU. Although costs seem to remain stable between posttreatment and 6MFU in the ICBT condition, we do not know whether this is because of treatment or other factors. The reason for using a WLC in the randomized controlled trial was that we judged the intervention research on stress-related disorders to still be in its early stages. In this context, the use of WLC has been suggested to be a viable option because it gives protection against basic threats to internal validity (eg, regression toward the mean and spontaneous remission) and reduces the risk of type-II error, which are central factors in the early phase of treatment development [53]. Furthermore, the WLC represents a realistic scenario, given that no established treatment guidelines exist for AD or ED and many suffering individuals never get access to psychological treatment [54].

A second limitation was that we relied on self-report data to obtain information about costs, which are prone to certain biases (eg, memory bias). Using self-report data to estimate costs in economic evaluations conducted together with randomized trials is, however, the most common procedure [55], and the TIC-P has demonstrated reasonable validity and reliability [39]. Moreover, there are indications of high convergence between self-report of health care visits and registry data [56].

A third issue is that of generalizability. In this study, the fact that the treatment was delivered via the internet likely improves generalizability, as it makes the results less dependent on factors such as regional differences in clinical practice and resource use (ie, patients were not tied to any specific setting). However, the high percentage of educated women in our sample limits generalizability to the full spectrum of individuals suffering from stress-related disorders. Even though the effect of gender on outcome in CBT has been found to be limited [57], and women with a moderate to high level of education tend to be overrepresented among people who seek health care for stress-related disorders (refer to, eg, the studies by Salomonsson et al [27], Heber et al [58], and Netterstrom et al [59]), more research on the cost-effectiveness of ICBT is needed using different types of patient samples and treatment settings.

Central strengths of this study were the randomized design, which enabled control over confounders; low attrition rates; and the use of both societal and health care perspectives in the health economic evaluation. Another central strength was that we used a clinical sample for which no past health economic evaluations have been conducted. We characterized heterogeneity through sensitivity analysis of the diagnostic subgroups AD and ED, which is often overlooked in health economic trials [60].

### Conclusions

In conclusion, ICBT for the stress-related disorders AD and ED is likely to be a cost-effective treatment compared with WLC. Most probably, ICBT leads to substantial improvements at no net societal costs, meaning that the treatment costs associated with administrating ICBT are likely to be quickly returned to society. In this trial, nearly two-thirds of patients who received ICBT were in remission after 12 weeks. Considering the scalability of this treatment and the low net costs, we believe that if ICBT is implemented in routine health care, it could play an important role in making effective treatment accessible to patients suffering from chronic stress in the form of AD and ED.

## Appendix

Multimedia Appendix 1 Supplementary material presenting estimation of costs, cost-utility analysis based on Quality-Adjusted Life Years, and additional results from sensitivity analyses.

## Abbreviations

6MFU: 6-month follow-up
AD: adjustment disorder
CBT: cognitive behavioral therapy
DSM-5: Diagnostic and Statistical Manual of Mental Disorders, 5th edition
ED: exhaustion disorder
EQ-5D: EuroQol 3L Questionnaire
ICBT: internet-based cognitive behavioral therapy
ICER: incremental cost-effectiveness ratio
ICUR: incremental cost-utility ratio
PSS: Perceived Stress Scale
QALY: quality-adjusted life year
SEK: Swedish Krona
TIC-P: Trimbos and Institute Medical Technology Assessment of Costs Questionnaire for Psychiatry
WLC: waitlist control
WTP: willingness to pay
