# Supplementary material for: Cost-Effectiveness of Therapist-Guided Internet-Based Cognitive Behavioral Therapy for Stress-Related Disorders: Secondary Analysis of a Randomized Controlled Trial
Source: J Med Internet Res. 2019 Sep 13;21(9):e14675. doi: 10.2196/14675 (PMC6788336; doi:10.2196/14675)
Supplement: Multimedia Appendix 1 [file jmir_v21i9e14675_app1.docx]

# Appendix 1

## Estimation of costs

| Table S1. Estimated costs for the most common types of direct and indirect health care services used by patients. | | |
| --- | --- | --- |
| Type of visit | Unit | Costs (US $) |
|  |  |  |
| General practitioner | Consultation | 231 |
| Company physician | Consultation | 138 |
| District nurse | Consultation | 77 |
| Physiotherapist | Consultation | 77 |
| Psychologist, private practice | Session | 115 |
| Psychologist, primary healthcare | Session | 103 |
| Psychiatrist | Consultation | 470 |
| Medical specialist (other) | Consultation | 367 |
| Home care | Hour | 34 |
| Alternative care | Session | 76 |
| Informal care (family, friends) | Hour | 10 |

Table S2. Per capita costs^a^ at the 6-month follow-up.

| Cost-domains | | | 6-month follow-up | |
| --- | --- | --- | --- | --- |
|  |  |  | ICBT^b^, n=47 | |
|  | | | Mean (SD) | Median |
| Direct medical | | | 244 (489) | 5 |
|  | Health care visits | | 240 (489) | 0 |
|  | Medication | | 4 (6) | 1 |
| Direct nonmedical | | | 54 (116) | 0 |
| Indirect nonmedical | | | 967 (1590) | 74 |
|  | | Unemployment | 286 (1119) | 0 |
|  | | Sick leave | 465 (1136) | 0 |
|  | | Work cutback | 167 (344) | 0 |
|  | | Domestic | 49 (106) | 2 |
| Gross total costs | | | 1266 (1845) | 333 |
| Intervention costs | | | —^c^ | — |
| Net total costs | | | 1266 (1845) | 333 |

^a^All costs are in US $, converted from the Swedish Krona.

^b^ICBT: Internet-based cognitive behavioral therapy.

^c^There were no intervention costs at the 6 month follow-up.

## Estimation of cost-utility using QALYs (Quality-Adjusted Life Years)

Figure S1. Cost-utility plane from a societal perspective (ie, including all direct and indirect medical and non-medical costs) comprising 5000 bootstrapped incremental cost-utility ratios comparing internet-based cognitive behavioral therapy to a wait-list control group over the 12-week treatment period using Quality-Adjusted Life Years (QALYs) as outcome, based on EQ-5D 3L (EuroQol) utilities. All costs are in US $, converted from the Swedish Krona.

Figure S2. Cost-utility acceptability curve from a societal perspective showing the probability of internet-based cognitive behavioral therapy (ICBT) being cost-effective compared with the wait-list control (WLC) at different willingness-to-pay ceilings, using Quality-Adjusted Life Years (QALYs) based on EQ-5D 3L (EuroQol) utilities.

## Sensitivity analysis: Cost-effectiveness of diagnostic subgroups

| Table S2. Means (SDs) of primary outcome measure and health-related quality of life for patients with adjustment disorder and exhaustion disorder respectively. | | | | | |
| --- | --- | --- | --- | --- | --- |
| Diagnostic group | Measure | Group | Pre, mean (SD) | Post, mean (SD) | 6MFU, mean (SD) |
|  | PSS | ICBT | 35.0 (7.1) | 23.9 (8.0) | 22.5 (8.5) |
| Adjustment disorder |  | WLC | 34.6 (5.6) | 30.8 (6.3) | —^a^ |
|  | Utility^b^ | ICBT | 0.86 (0.08) | 0.90 (0.07) | 0.90 (0.08) |
|  |  | WLC | 0.86 (0.8) | 0.89 (0.10) | — |
|  | PSS | ICBT | 39.7 (6.2) | 24.6 (9.4) | 21.2 (6.6) |
| Exhaustion disorder |  | WLC | 38.4 (8.4) | 35.7 (8.6) | —^a^ |
|  | Utility^b^ | ICBT | 0.77 (0.14) | 0.84 (0.10) | 0.87 (0.10) |
|  |  | WLC | 0.78 (0.13) | 0.80 (0.11) | — |

Pre: Pretreatment assessment; Post: Posttreatment assessment; 6MFU: 6-month follow-up; PSS: 14-item Perceived Stress Scale; ICBT: Internet-based cognitive behavioral therapy; WLC: wait-list control.

^a^Not applicable.

^b^Utility scores are based on EQ-5D 3L (EuroQol) health states.

| Table S3. Per capita costs (US $) at each assessment point for patients with adjustment disorder, presented as mean (SD), median. | | | | | | | | | |  |
| --- | --- | --- | --- | --- | --- | --- | --- | --- | --- | --- |
| Cost-domains |  |  | Pretreatment | |  | Posttreatment | |  | 6MFU |  |
|  |  |  | ICBT, n=27 | WLC, n=26 |  | ICBT, n=27 | WLC, n=24 |  | ICBT, n=26 |  |
| Direct medical |  |  | 134 (317), 1 | 116 (209), 1 |  | 99 (167), 6 | 204 (210), 231 | | 226 (408), 5 |  |
| Healthcare |  |  | 139 (315), 0 | 114 (207), 0 |  | 94 (166), 0 | 201 (209), 231 | | 222 (481), 0 |  |
| Medication |  |  | 4 (8), 1 | 2 (4), 0 |  | 6 (19), 1 | 3 (5), 1 |  | 4 (6), 1 |  |
| Direct non-medical |  |  | 61 (104), 0 | 103 (120), 76 | | 89 (134), 20 | 118 (192), 10 | | 58 (136), 0 |  |
| Indirect non-medical |  |  | 601 (1172), 181 | 442 (1345), 52 | | 442 (923), 136 | 487 (894), 171 | | 723 (1501), 3 |  |
| Unemployment |  |  | 278 (1006), 0 | 0 (0), 0 |  | 130 (674), 0 | 0 (0), 0 |  | 309 (1092), 0 |  |
| Sick leave |  |  | 20 (76), 0 | 267 (1212), 0 | | 149 (678), 0 | 259 (840), 0 |  | 185 (797), 0 |  |
| Work cutback |  |  | 220 (344), 30 | 117 (269), 0 |  | 137 (176), 75 | 161 (269), 0 |  | 176 (377), 0 |  |
| Domestic |  |  | 82 (241), 7 | 57 (126), 6 |  | 26 (51), 6 | 57 (136), 7 |  | 53 (121), 0 |  |
| **Gross total costs** |  |  | 796 (295), 1296 | 661 (1417), 231 | | 630 (1036), 314 | 800 (960), 604 | | 1006 (1703), 225 |  |
| Intervention costs |  |  | —^a^ | — |  | 182 (65), 186 | — |  | — |  |
| **Net total costs** | |  | 796 (295), 1296 | 661 (1417), 231 | | 812 (1063), 469 | 800 (960), 604 | | 1006 (1703), 225 |  |
| 6MFU: 6-month follow-up; ICBT: Internet-based cognitive behavioral therapy; WLC: wait-list control.  ^a^Not applicable.   \| Table S4. Per capita costs (US $) at each assessment point for patients with exhaustion disorder, presented as mean (SD), median. \| \| \| \| \| \| \| \| \| \| \| --- \| --- \| --- \| --- \| --- \| --- \| --- \| --- \| --- \| --- \| \| Cost-domains \|  \|  \| Pretreatment \| \|  \| Posttreatment \| \|  \| 6MFU \| \|  \|  \|  \| ICBT, n=23 \| WLC, n=24 \|  \| ICBT, n=22 \| WLC, n=24 \|  \| ICBT, n=20 \| \| Direct medical \|  \|  \| 249 (318), 115 \| 532 (704), 315 \|  \| 206 (314), 231 \| 497 (1170), 176 \| \| 281 (521), 8 \| \| Healthcare \|  \|  \| 216 (251), 115 \| 527 (704), 305 \| \| 253 (314), 231 \| 490 (1170), 165 \| \| 277 (519), 0 \| \| Medication \|  \|  \| 34 (121), 4 \| 5 (5), 4 \|  \| 6 (8), 2 \| 7 (8), 5 \|  \| 4 (7), 1 \| \| Direct non-medical \|  \|  \| 102 (136), 40 \| 221 (632), 0 \|  \| 64 (128), 0 \| 101 (156), 0 \|  \| 52 (88), 0 \| \| Indirect non-medical \|  \|  \| 1718 (1506), 1151 \| 1694 (1685), 931 \| \| 1428 (1592), 839 \| 1601 (1539), 992 \| \| 1329 (1702), 666 \| \| Unemployment \|  \|  \| 0 (0), 0 \| 167 (820), 0 \|  \| 245 (1150), 0 \| 0 (0), 0 \|  \| 270 (1206), 0 \| \| Sick leave \|  \|  \| 821 (1346), 0 \| 1043 (1513), 0 \| \| 792 (1079), 100 \| 989 (1531), 0 \| \| 852 (1418), 0 \| \| Work cutback \|  \|  \| 628 (1346), 201 \| 332 (667), 40 \| \| 303 (592), 0 \| 472 (562), 295 \| \| 165 (313), 0 \| \| Domestic \|  \|  \| 269 (513), 128 \| 151 (233), 76 \| \| 87 (93), 63 \| 140 (165), 58 \| \| 43 (88), 16 \| \| **Gross total costs** \|  \|  \| 2070 (1528), 1651 \| 2446 (2304), 2007 \| \| 1752 (1811), 1218 \| 2199 (2233), 1329 \| \| 1663 (2003), 995 \| \| Intervention costs \|  \|  \| —^a^ \| — \|  \| 228 (85), 226 — \| \|  \| — \| \| **Net total costs** \| \|  \| 2070 (1528), 1651 \| 2446 (2304), 2007 \| \| 1980 (1831), 1412 \| 2199 (2233), 1329 \| \| 1663 (2003), 995 \|   6MFU: 6-month follow-up; ICBT: Internet-based cognitive behavioral therapy; WLC: wait-list control.  ^a^Not applicable. | | | | | | | | | | |


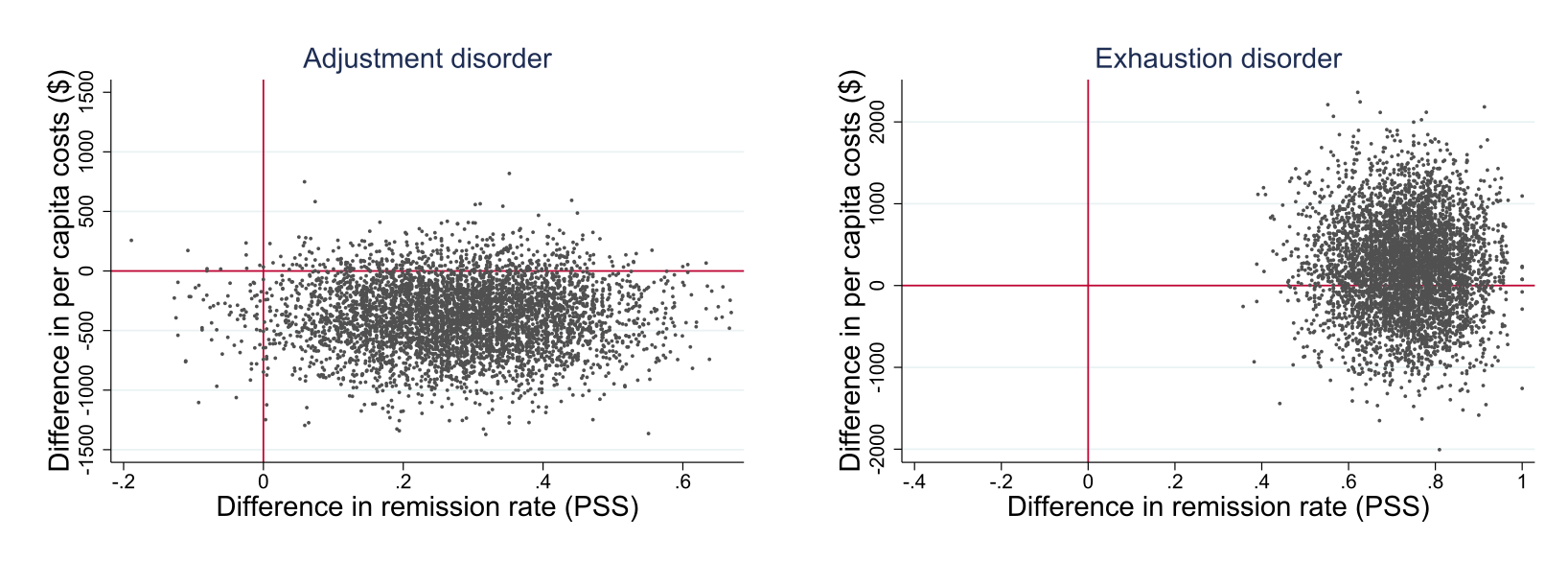


Figure S3. Cost-effectiveness planes from a societal perspective comprising 5000 bootstrapped incremental cost-effectiveness ratios comparing internet-based cognitive behavioral therapy with a wait-list control group over the 12-week treatment period, stratified by diagnosis, ie, adjustment disorder (left plane) and exhaustion disorder (right plane). Remission was operationalized as proportion of patients who made a clinically significant improvement on the 14-item Perceived Stress Scale (PSS). All costs are in US $, converted from the Swedish Krona.

## Sensitivity analysis: Higher cost-estimate of domestic work

Figure S4. Cost-effectiveness plane from a societal perspective comprising 5000 bootstrapped incremental cost-effectiveness ratios (ICERs) comparing internet-based cognitive behavioral therapy with a wait-list control group over the 12-week treatment period. The ICERs were based on a sensitivity analysis in which a higher estimate of costs for domestic work and informal care was used, US $19 as opposed to US $10 used in the main analysis. Remission was operationalized as proportion of patients who made a clinically significant improvement on the 14-item Perceived Stress Scale (PSS). All costs are in US $, converted from the Swedish Krona.

## Sensitivity analysis: Removed costs for domestic work

Figure S5. Cost-effectiveness plane from a societal perspective, excluding costs for domestic work and informal care from total net costs. The plane comprises 5000 bootstrapped incremental cost-effectiveness ratios comparing internet-based cognitive behavioral therapy with a wait-list control group over the 12-week treatment period. Remission was operationalized as proportion of patients who made a clinically significant improvement on the 14-item Perceived Stress Scale (PSS). All costs are in US $, converted from the Swedish Krona.

## Sensitivity analysis: Removed costs for work cutback

Figure S6. Cost-effectiveness plane from a societal perspective, excluding costs for work cutback from total net costs. The plane comprises 5000 bootstrapped incremental cost-effectiveness ratios comparing internet-based cognitive behavioral therapy with a wait-list control group over the 12-week treatment period. Remission was operationalized as proportion of patients who made a clinically significant improvement on the 14-item Perceived Stress Scale (PSS). All costs are in US $, converted from the Swedish Krona.

Figure S7. Cost-effectiveness acceptability curve from a societal perspective showing the probability of internet-based cognitive behavioral therapy (ICBT) being cost-effective compared with the wait-list control (WLC) at different willingness-to-pay ceilings, in a scenario in which costs for work cutback are excluded from total net costs. Remission was operationalized as proportion of patients who made a clinically significant improvement on the 14-item Perceived Stress Scale (PSS).

## Sensitivity analysis: Doubling costs for work cutback

Figure S8. Cost-effectiveness plane from a societal perspective comprising 5000 bootstrapped incremental cost-effectiveness ratios (ICERs) comparing internet-based cognitive behavioral therapy with a wait-list control group over the 12-week treatment period. The ICERs were based on a sensitivity analysis in which costs for work cutback were doubled. Remission was operationalized as proportion of patients who made a clinically significant improvement on the 14-item Perceived Stress Scale (PSS). All costs are in US $, converted from the Swedish Krona.
